# Acute gastric volvulus associated with wandering spleen in an adult treated laparoscopically after endoscopic reduction: a case report

**DOI:** 10.1186/s40792-016-0175-0

**Published:** 2016-05-24

**Authors:** Jiro Omata, Katsuyuki Utsunomiya, Yoshiki Kajiwara, Risa Takahata, Nobuo Miyasaka, Hidekazu Sugasawa, Naoko Sakamoto, Yoji Yamagishi, Makiko Fukumura, Daiki Kitagawa, Mitsuhiko Konno, Yasushi Okusa, Michinori Murayama

**Affiliations:** Department of Surgery, Japan Self-Defense Force Central Hospital, 1-2-24 Ikejiri, Setagaya-ku, Tokyo 154-8532 Japan; Department of Surgery, KKR Mishuku Hospital, 5-33-12 Kamimeguro, Meguro-ku, Tokyo 153-0051 Japan; Department of Surgery, National Defense Medical College, 3-2 Namiki, Tokorozawa, Saitama 359-8513 Japan; Medical Office, Ministry of Defense, 5-1 Ichigayahonmura, Shinjuku-ku, Tokyo 162-8801 Japan; Department of Gastroenterology, KKR Mishuku Hospital, 5-33-12 Kamimeguro, Meguro-ku, Tokyo 153-0051 Japan

**Keywords:** Gastric volvulus, Wandering spleen, Laparoscopic surgery, Gastropexy, Endoscopic reduction

## Abstract

A 43-year-old female was referred to our hospital for sudden onset of abdominal pain, fullness, and vomiting. Physical examination revealed abdominal distension with mild epigastric tenderness. Abdominal radiography showed massive gastric distension and plain computed tomography (CT) a markedly enlarged stomach filled with gas and fluid. A large volume of gastric contents was suctioned out via a nasogastric (NG) tube. Contrast-enhanced CT showed a grossly distended stomach with displacement of the antrum above the gastroesophageal junction, and the spleen was dislocated inferiorly. Upper gastrointestinal (GI) series showed the greater curvature to be elevated and the gastric fundus to be lower than normal. Acute mesenteroaxial gastric volvulus was diagnosed. GI endoscopy showed a distortion of the gastric anatomy with difficulty intubating the pylorus. Various endoscopic maneuvers were required to reposition the stomach, and the symptoms showed immediate and complete solution. GI fluoroscopy was performed 3 days later. Initially, most of the contrast medium accumulated in the fundus, which was drawn prominently downward, and then began flowing into the duodenum with anteflexion. Elective laparoscopic surgery was performed 1 month later. The stomach was in its normal position, but the fundus was folded posteroinferiorly. The spleen attached to the fundus was normal in size but extremely mobile. We diagnosed a wandering spleen based on the operative findings. Gastropexy was performed for the treatment of gastric volvulus and wandering spleen. The patient remained asymptomatic, and there was no evidence of recurrence during a follow-up period of 24 months. This report describes a rare adult case of acute gastric volvulus associated with wandering spleen. Because delay in treatment can result in lethal complications, it is critical to provide a prompt and correct diagnosis and surgical intervention. We advocate laparoscopic surgery after endoscopic reduction because it is a safe and effective procedure with lower invasiveness.

## Background

Gastric volvulus is a rare clinical condition presenting as an acute abdomen. Gastric volvulus is characterized by abnormal laxity or absence of the supporting ligaments associated with various congenital or acquired conditions, leading to twisting of all or part of the stomach that may obstruct the gastric cavity [1–3]. Such rotation of the stomach can occur either along its horizontal axis, which is called organoaxial volvulus, or along its vertical axis, which is called mesenteroaxial volvulus [1–3]. A complete volvulus potentially leads to strangulation, which may result in ischemia, necrosis, and perforation [1, 2]. Wandering spleen is another rare condition characterized by excessive splenic mobility and displacement from the spleen’s original position to another location caused by the abnormal laxity or absence of ligaments that would normally keep the spleen immobile [4, 5]. Although both gastric volvulus and wandering spleen are caused by the common abnormality of an underdeveloped dorsal mesentery, the association of gastric volvulus and wandering spleen is extremely rare. To the best of our knowledge, of the 52 cases described in the literature through the end of 2015, most were children, and only 3 cases were adults (Table 1) [6–22]. Because mortality rates of acute gastric volvulus reportedly range from 30 to 50 % with the major cause of death being strangulation [1, 2], it is important to provide a rapid and correct diagnosis and surgical management. We herein present a rare case of acute gastric volvulus associated with wandering spleen treated with laparoscopic surgery after successful endoscopic reduction.

**Table 1:** Reported adult cases of acute gastric volvulus associated with wandering spleen

Case	1	2	3	Present case
Year	2006	2013	2013	2016
Age (years)	67	28	22	43
Sex	M	F	M	F
Patient history	Schizophrenia	None	Wilson disease	None
Symptoms	Distension, vomiting	Pain, nausea, vomiting	Pain	Pain, distension, vomiting
Previous episode	Multiple	Multiple	Multiple	Multiple
Volvulus type	Mesenteroaxial	Mesenteroaxial	Mesenteroaxial	Mesenteroaxial
Decompression	NG tube	NG tube	NG tube	NG tube, upper GI endoscopy
Predisposing factor	Wandering spleen	Wandering spleen	Wandering spleen	Wandering spleen
Strategy	Elective-LS	Exploratory-OS	Exploratory -LS	Elective-LS
Treatment	Gastropexy	Gastropexy	No surgical intervention	Gastropexy
Complication	None	Gastric ischemia	None	None
Follow-up	ND	2 months	6 months	24 months
Reference	6	7	8	–

*M* male, *F* female, *NG* nasogastric, *GI* gastrointestinal, *LS* laparoscopic surgery, *OS* open surgery, *ND* not described

## Case presentation

A 43-year-old female presented to our emergency room complaining of abdominal pain, fullness, and vomiting. The pain was acute in onset, colicky, and continuous, and these symptoms gradually worsened. Just prior to the onset of these symptoms, she had been completely well and eaten dinner as usual. Her medical history was unremarkable. Because she had periodically experienced similar symptoms since childhood, she had undergone detailed examinations several times, but the origin of the symptoms was not identified. The patient was conscious and oriented and had a pulse of 77 beats per minute, a blood pressure of 154/92 mmHg, a body temperature of 36.5 °C, and an oxygen saturation of 98 % on room air. Physical examination revealed abdominal distension with mild epigastric tenderness. Laboratory results were as follows: white blood cell count 12,820/mL (normal, 4000–9000) and serum C-reactive protein concentration 0.48 mg/dL (<0.3) and liver functions, renal functions, creatine kinase, and lactate dehydrogenase within normal ranges.

**Fig. 1:**
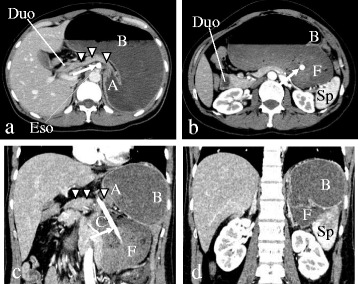
Contrast-enhanced abdominal CT findings. The *arrow* shows the NG tube inserted into the stomach. Axial CT scan at the abdominal esophagus level (**a**) demonstrates the grossly enlarged stomach with resultant displacement of the gastric antrum (*A* and *arrowheads*) above the abdominal esophagus. More caudal axial CT scan (**b**) and coronal CT images (**c**, **d**) reveal the stomach to be twisted mesenteroaxially, with the antrum (*A*) positioned higher than the fundus (*F*). CT findings (**b**, **d**) show the normal-sized spleen positioned inferiorly toward the left kidney as compared to its normal position. *CT* computed tomography, *NG tube* nasogastric tube, *A* antrum, *B* body, *C* cardia, *Duo* duodenum, *Eso* esophagus, *F* fundus, *Sp* spleen

**Fig. 2:**
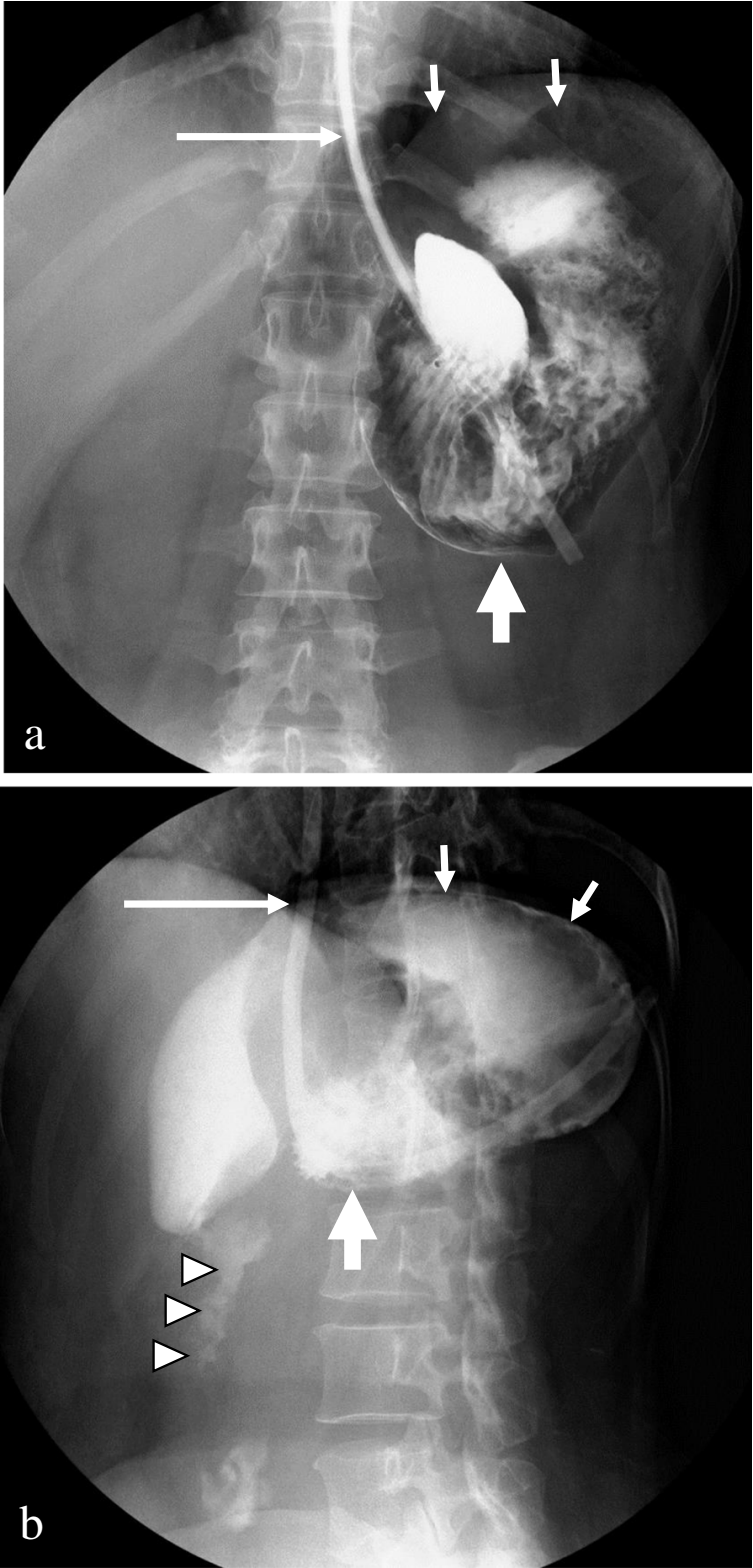
Upper GI contrast radiogram. Upper GI series in the supine position (**a**) and a lateral view obtained with the patient standing upright (**b**) demonstrate a high greater curvature (*short arrows*), with the greater curvature crossing the esophagus (*long arrow*), the pylorus pointing downward (*arrowheads*), and that the gastric fundus is lower than normal (*thick arrow*). *GI* gastrointestinal

**Fig. 3:**
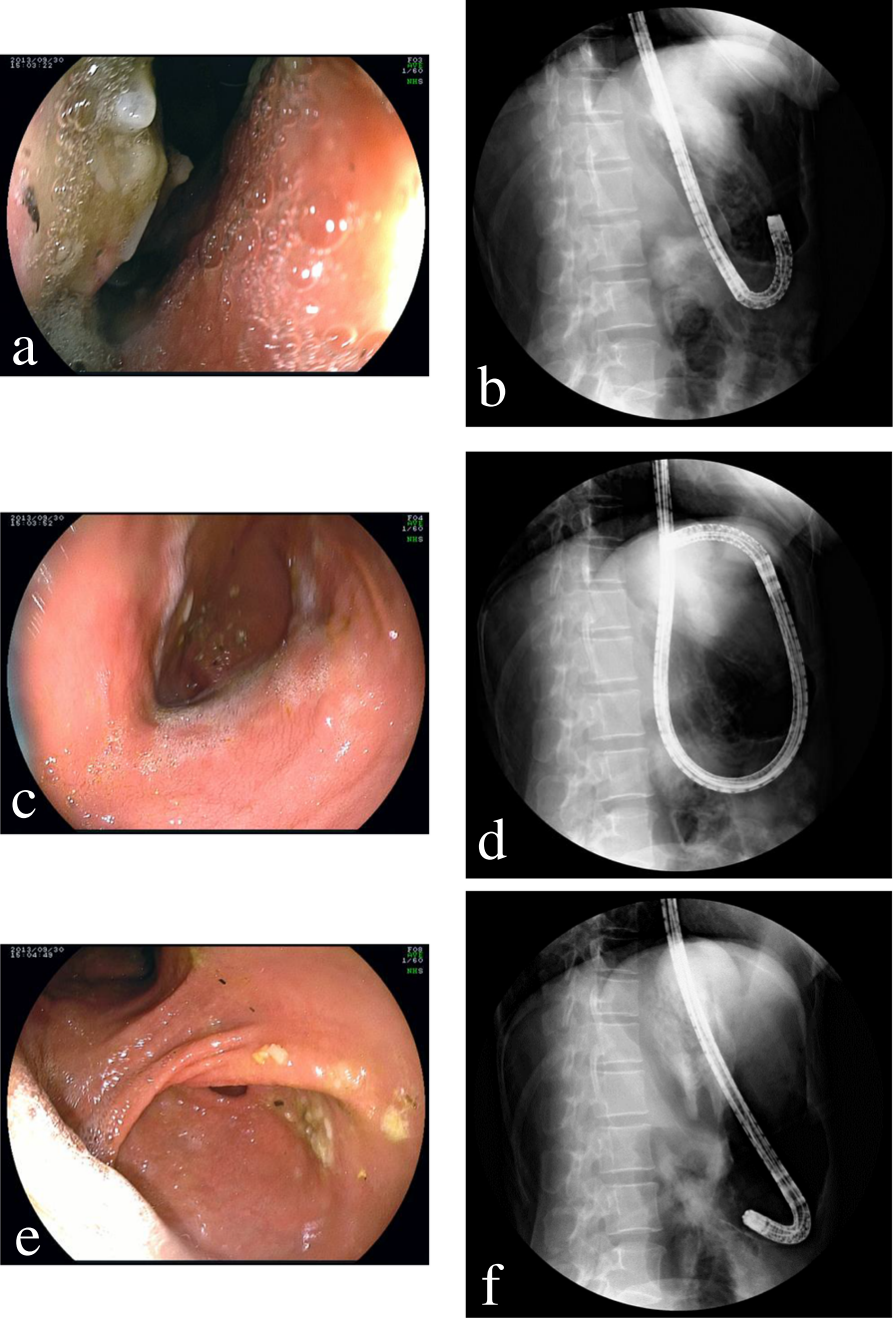
Endoscopic reduction of gastric volvulus using radiographic imaging. **a** Upper GI endoscopy shows the dilated stomach containing residual food and fluid (GI image of **a** is **b**). **c** The endoscope cannot be passed through the pylorus (GI image of **c** is **d**). **e** With various endoscopic maneuvers, such as clockwise rotation and pulling the GI endoscope back, the gastric volvulus is reduced and the endoscope passes through the pylorus into the duodenum (GI image of **e** is **f**). *GI* gastrointestinal

Abdominal radiography showed a massively distended viscus in the upper abdomen. A plain computed tomography (CT) scan demonstrated the stomach to be markedly distended and filled with gas and fluid. A nasogastric (NG) tube was inserted with difficulty. A large volume of gastric contents was suctioned out via the NG tube, promptly relieving the abdominal pain. Further, plain abdominal radiography showed the stomach to be markedly dilated with a double air fluid level when the patient was standing. The patient underwent a contrast-enhanced CT, which revealed a grossly enlarged stomach with resultant displacement of the gastric antrum above the gastroesophageal junction and a normal-size spleen positioned inferiorly toward the left kidney as compared to its normal anatomical location, and there was no evidence of ischemia, infarction, or perforation of the abdominal organs (Fig. 1). Upper gastrointestinal (GI) series through the NG tube showed an elevated greater curvature, with the greater curvature crossing the esophagus, the pylorus pointing downward, and the gastric fundus in a lower position than normal (Fig. 2). These findings pointed to a diagnosis of acute mesenteroaxial gastric volvulus. Upper GI endoscopy revealed distortion of the gastric anatomy with difficulty intubating the pylorus (Fig. 3). Employing various endoscopic maneuvers such as clockwise rotation and pulling the endoscope back, we succeeded in repositioning the stomach and GI endoscopy then passed through the pylorus into the duodenum. Abdominal radiography confirmed gastric volvulus reduction. The patient’s symptoms showed immediate and complete solution after this reduction, and her subsequent course was uneventful. After a 3-day recovery period, the patient was performed a further GI fluoroscopy with contrast medium. Initially, most of the contrast medium accumulated in the fundus, which was drawn prominently downward, and then began flowing into the duodenum with anteflexion. The patient was discharged from the hospital, and elective surgery was planned for 1 month later.

**Fig. 4:**
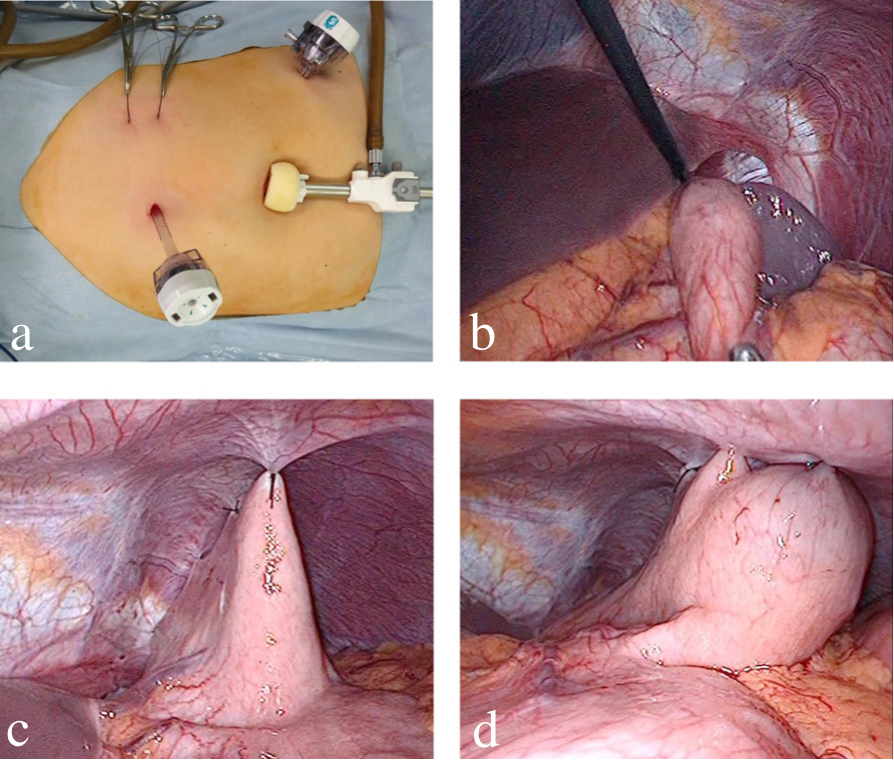
Laparoscopic surgery for gastric volvulus. **a** The placement of three trocars and two small incisions made for gastropexy. **b** The fundus is folded posteroinferiorly, and the spleen is attached to the fundus which is freely mobile. The operative findings confirm the diagnosis of wandering spleen. **c** Phrenofundopexy is performed to prevent lowering of the fundus and to keep the spleen fixed in the left upper abdomen. **d** Anterior gastropexy is performed to prevent the stomach from twisting

Laparoscopic surgery was performed under general anesthesia. A 12-mm port was inserted at the umbilicus and two 5-mm ports were placed in the epigastrium and the lower left abdomen (Fig. 4a). There was no evidence of hiatal hernia or diaphragmatic defect. The stomach was in its normal anatomical position, but the fundus was folded posteroinferiorly. The spleen attached to the fundus was normal in size but hyper-mobile (Fig. 4b). The surrounding splenic ligaments other than the gastrosplenic ligament were absent. Therefore, we diagnosed a wandering spleen based on the operative findings. The lower than the normal position of the fundus was attributed to the abnormal gastrophrenic ligament which was probably associated with wandering spleen. We performed phrenofundopexy and anterior gastropexy, laparoscopically. The fundus at the greater curvature of the stomach was fixed to the diaphragm with five interrupted nonabsorbable sutures in order to prevent the fundus from being folded and to keep the spleen fixed in the left upper abdomen (Fig. 4c). The upper body was anchored to the anterior abdominal wall with two interrupted absorbable sutures in order to prevent the stomach from twisting (Fig. 4d).

The postoperative period was uneventful. The contrast medium used for GI radiography on the fourth day after surgery showed good passage without pooling in the fundus, and the patient was discharged 5 days postoperatively. She remained asymptomatic, and there has been no evidence of gastric volvulus or wandering spleen on the radiological images obtained to date, 24 months after the operation.

### Discussion

It is critical to make a prompt and precise diagnosis in order to avoid the potentially fatal conditions associated with prolonged volvulus such as ischemia, necrosis, and perforation of the stomach. Since the diagnosis is difficult to make based on clinical features alone, several imaging studies may be employed to facilitate the diagnosis of gastric volvulus and coexisting disorders. Radiography, GI fluoroscopy, and CT are the effective modalities most frequently employed [3]. Radiography shows a massive distended stomach with air in supine position and a double air-fluid level in upright position. Upper GI fluoroscopy can be performed to evaluate rotation of the stomach and the passage of ingested contrast material into the duodenum. CT is especially reliable for diagnosing acute gastric volvulus, consequent critical complications, and factors triggering the onset. GI endoscopy is, however, unreliable for the diagnosis of latent gastric volvulus [23]. With the advanced diagnosis and management now available, the mortality rate of acute gastric volvulus has decreased to 15–20 % [24].

The radiological findings in our case demonstrated a mesenteroaxial gastric volvulus. Mesenteroaxial gastric volvulus results from rotation of the stomach around the lesser and greater curvatures, with resultant displacement of the antrum above the gastroesophageal junction. Mesenteroaxial volvulus usually occurs partially and intermittently, and obstruction and strangulation are less common [1, 3]. The patient had complained of intermittent dyspeptic pain and abdominal fullness after meals, which was the chronic symptoms with a high recurrence rate (64 %) [23], and acute-on-chronic gastric volvulus with Borchardt’s triad was occurred. The radiological and surgical findings of our present patient included the fundus being located posteroinferiorly as compared to its normal position and a wandering spleen attached to the fundus. Since some patients of wandering spleen are completely asymptomatic, the diagnosis may be made incidentally by routine physical examination or imaging [17]. A preoperative diagnosis of wandering spleen reportedly accounts for only approximately 50 % of cases [12]. We were not able to diagnose a wandering spleen preoperatively in this case. Presumably, in our case, the etiology of gastric volvulus would have been acquired laxity of the gastric ligaments, possibly associated with a wandering spleen, allowing the resultant rotation of the stomach due to the weight of gastric contents accumulated in the fundus along the short axis when the stomach was full, leading to volvulus [10].

The treatment of gastric volvulus involves decompression of the stomach, reduction of the volvulus, gastropexy, and correction of the underlying cause [1, 25]. NG tube placement is a brief and effective procedure for decompression of the stomach. Upper GI endoscopy is the most effective method of achieving decompression and reduction of the stomach in the emergency setting, rapidly leading to a marked improvement of the patient’s condition [26–28]. Definitive treatment of gastric volvulus includes gastropexy and correction of the associated predisposing factors. It merits emphasis that correction of predisposing factors and gastric fixation procedures is required to prevent volvulus recurrence. Recent reports have documented the prevention of gastric volvulus by percutaneous endoscopic gastropexy with wide fixation of the stomach as a means of avoiding recurrence [28, 29]. This may be a feasible technique for high-risk patients because of its minimal invasiveness, but long-term studies are needed. Definitive treatments such as gastropexy, splenopexy, hernia reduction, and diaphragmatic hernia and esophageal hiatus repairs have been performed laparoscopically, for both acute and chronic conditions [24–26, 30]. Laparoscopic surgery is reportedly a safe and effective procedure, with lower morbidity rate and a significantly shorter hospital stay than laparotomy [30]. Moreover, laparoscopy yields an accurate etiologic diagnosis, and like laparotomy, several therapeutic options are available intraoperatively [12, 16, 24, 25]. In our patient, after endoscopic maneuvering to reduce acute symptoms, elective laparoscopic gastropexy was performed. Phrenofundopexy was performed to prevent lowering of the fundus and keep the spleen fixed in the left upper abdomen, and anterior gastropexy was performed to prevent the stomach from rotating. The pitch between the sutures was about 2.5 cm to prevent an internal herniation. In general, gastropexy in addition to splenopexy is recommended in the case of gastric volvulus with wandering spleen. The gastrosplenic ligament of the patient worked to localize wandering spleen around the left upper quadrant and to prevent torsion of splenic vessels. Because the spleen was closely fixed between the stomach and abdominal wall by the gastrosplenic ligament and gastropexy procedure, splenopexy was not performed for the purpose of the correction of associated predisposing factors. Moreover, splenopexy is the recommended procedure of choice to prevent future splenic torsion when wandering spleen is present at surgery [4, 16]. Approximately 65 % of patients with an acute presentation are asymptomatic prior to the occurrence of splenic torsion and infarction [4, 8]. However, splenopexy was not performed in this case, because it was unlikely to be torsion of the vascular pedicle owing to the presence of gastrosplenic ligament and the fixation of the spleen to the abdominal wall [6, 7, 9, 12]. The patient remained asymptomatic, and there has been no evidence of gastric volvulus recurrence or wandering spleen.

## Conclusions

This report describes a rare adult case of acute gastric volvulus associated with wandering spleen. Because delay in treatment may lead to fatal complications, it is critical to provide a prompt and precise diagnosis and surgical management. We recommend laparoscopic surgery after endoscopic reduction because it is a safe and effective procedure, with the advantages of various surgical techniques used previously and lower invasiveness.

## Abbreviations

CT: computed tomography
GI: gastrointestinal
NG: nasogastric
